# Incidence, Pattern, and Severity of Acute Respiratory Infections among Infants and Toddlers of a Peri-Urban Area of Delhi: A 12-Month Prospective Study

**DOI:** 10.1155/2014/165152

**Published:** 2014-09-21

**Authors:** Sneha P. Walke, Ranjan Das, Anita Shankar Acharya, Harish K. Pemde

**Affiliations:** ^1^BMC, Gorai Mhada Dispensary, Borivali West, Mumbai, Maharashtra 401107, India; ^2^Department of Community Medicine, Lady Hardinge Medical College & Associated Hospitals, New Delhi 110001, India; ^3^Department of Paediatrics, Lady Hardinge Medical College & Associated Hospitals, New Delhi 110001, India

## Abstract

Acute respiratory infections (ARIs) in spite of being the single most important under-five morbidity have not been studied adequately in peri-urban settings in India. We conducted this study prospectively on a cohort of 106 children in a peri-urban area of Delhi. The overall 2-week prevalence of all types of ARI was 34.3%. Annual combined incidence of all types of ARI was 7.9 episodes/100 child-weeks; while that for no pneumonia, cough, and cold, pneumonia, and otitis media was 7.1, 0.85, and 0.09 epi/100 ch-wks, respectively. Incidence of ARI was higher in infancy (9.4 epi/100 ch-wks) as compared to toddlers (7.0 epi/100 ch-wks). Pneumonia incidence was higher among boys (0.9 epi/100 ch-wks as compared to 0.6 for girls) and the highest in infants under 2 months of age (1.09 epi/100 ch-wks; *P* < 0.01). Incidence of severe pneumonia was roughly one-tenth that of pneumonia. Incidence of both ARI and pneumonia peaked in spring and autumn. Mothers of infants, zespecially those under 2 months of age, need to be made aware of ARI/pneumonia and IEC campaigns may be aired more intensively keeping their peak season in mind.

## 1. Introduction

Acute respiratory infections (ARIs) are important under-five morbidities causing lots of discomfort, frequent visits to a healthcare provider, admission to an indoor facility, and even mortality [1, 2]. India's Reproductive and Child Health programme has components for the prevention and control of ARI since 1992 [3]. ARIs, however, still continue to be the single largest contributor of childhood morbidity and mortality with estimated 3–5 episodes every year, 4 million pneumonias, and 1 million deaths [4–6]. Estimates also indicate that 30–50% of OPD attendance and 20–40% of hospital admissions may be attributed to ARI and pneumonia [7].

For a disease of such magnitude and severity, it is important that all aspects of its epidemiology have to be known. Yet unfortunately, there are few studies reporting the detailed epidemiology of ARIs. Of the few studies that exist, very few studies have been conducted in peri-urban communities and fewer involved active surveillance done prospectively by qualified and trained health practitioners. Besides, most studies are 1 to 2 decades old [8–17]. In view of above, a prospective study to find out the current epidemiology of ARIs among infants and toddlers, the most affected age group, was planned. We report here the incidence, prevalence, clinical pattern, severity, seasonal pattern, and gender differences.

## 2. Methods

This community-based follow-up study was conducted in 2011-12 at Mehrauli, a peri-urban field practice area attached to the Department of Community Medicine of a tertiary care teaching hospital of New Delhi. Optimum sample size was worked out to be 106 children or 5512 child-weeks of exposure. Owing to logistics needed for the fortnightly follow-up, the study was restricted to one of the wards selected randomly (Ward 4). Cut-off age for enrollment of children was kept as 24 months so as to ensure a follow-up of 12 months before child ceased to be toddler. The only inclusion criterion was parents' willingness to let their children participate, while exclusion criteria were two-fold: (i) child suffering from any chronic/severe illness and (ii) family of child not a permanent resident of Ward 4. Ethical clearance was granted by institutional ethics committee. Written informed consent was obtained from the parent or the caregiver of the study subject.

Our sampling frame was constituted of 264 under-two children identified through a house-to-house survey one month prior to the study. Of these, 23 were not eligible for being included in the study (1 had thalassemia, 1 had nephrotic syndrome, 1 had very low birth weight, and 20 were temporary residents). Study subjects were selected randomly from the remaining 241 eligible subjects. Three parents refused to let their wards participate and were replaced by another eligible child residing closest to the refusal.

Assessment for presence of ARI was done through history and physical examination [18]. History regarding presence of ARI currently or in the past fortnight was elicited from a responsible caregiver and recorded on MICS proforma of UNICEF [19]. All children, whether or not their parents reported a current/past bout of ARI, were assessed clinically by a qualified trained medical practitioner on the designated day. Additional information on type of ARI, its severity, other accompanying symptoms, and so forth was assessed and recorded in a separate interview schedule validated by a faculty from paediatrics department.

Background information on the physical, biological, and sociodemographic environment as well as the general health status of the child was noted. In context of physical environment, overcrowding, indoor air pollution, environmental tobacco smoke, and so forth were specifically looked into. History of breast feeding, immunization, and exposure to ARI from siblings was assessed as part of the biological environment. Education, occupation, income, and social class formed the main stay of our sociodemographic assessment. General health status of the child was assessed through the standard general physical examination. Standard methods were used for recording weight and length. Variations in clinicoenvironmental parameters were noted in every follow-up visit.

Information collected on interview schedules was cross-checked for completeness and correctness by supervisors and a database was created using Microsoft Excel software. Double data entry was done for ensuring maximum accuracy and minimizing errors. Statistical analysis was done using SPSS version 13. Incidence density of all ARIs has been described in terms of mean number of episodes per 100 child-weeks of exposure. Chi-square test has been used to detect statistical significance at 5% level of significance.

## 3. Results

A total of 2752 contacts were made with 67 boys and 39 girls at their residences. One enrolled subject went out of town for 2 months, thus reducing our observation period by 8 child-weeks to 5503 child-weeks of exposure. Thus, an overall response rate of 98.4% was achieved.  Table 1 shows the sociodemographic and environmental characteristics of families of study subjects. Four-fifth of study subjects were from middle income group and the remaining from high income families [20].

A little over 5% of the mothers were illiterate and 32.1% had less than five years of schooling. Nearly 70% of subjects belonged to joint families. Close to half of the subjects lived in overcrowded (47%) or ill-ventilated (21%) houses, and 14% of households did not have a separate kitchen and 18% were using biomass fuels in addition to liquefied petroleum gas (LPG). Besides, 45% of study subjects were exposed to environmental tobacco smoke (ETS). One-fourth of subjects were having mild-to-moderate protein energy malnutrition (PEM).

Health assessment based on history as well as physical examination revealed that ARI was the commonest illness (437 episodes in 105 children) followed by diarrhea (253 episodes in 85 individuals) and fever (69 episodes in 48 individuals). Prevalence of ARI, diarrhea, and fever was thus found to be 34.5, 19.9, and 5.4 percent, respectively. Only 1 child did not seem to suffer from any type of ARI during the study period, 105 (99.1%) children suffered from no pneumonia, cough, and cold (alone or in combination with other types of ARI), 38 (35.8%) had pneumonia, and 5 (4.7%) had otitis media. Of the 437 ARI episodes, 390 (89.2%) affected the upper respiratory tract only, 47 (10.7%) were pneumonias, and 5 (1.1%) were otitis media. Out of those children suffering from ARI, proportions of children suffering from 1 to 3, 4 to 5, and 6 to 7 episodes of ARI were 35.2, 45.7, and 19.1 percent, respectively. Of the 38 children who suffered from pneumonia, 9 (23.7%) had two episodes. Three children had to be hospitalized and all of them had severe pneumonia. No mortality was encountered during the study period. The mean duration of URI was 6.16 days (range: 4–14 days) as compared to 11.65 days for LRI (range: 7–19 days).

Table 2 shows the incidence of ARI according to age. The overall incidence of ARI (all types) was found to be 7.9 episodes/100 child-weeks of exposure; incidence was higher in infancy (9.4 episodes/100 child-weeks) as compared to toddlers (7.0 episodes/per 100 child-weeks). Incidence of pneumonia was found to be 0.8 episodes/100 child-weeks (1.1 in infancy and 0.7 in toddlers). Highest incidence of pneumonia was seen in children less than 2 months of age (1.7 episodes/100 child-weeks). Incidence of no pneumonia, cough, and cold was nearly constant throughout infancy, decreasing in the second and third years of life (lowest incidence of 3.3 episodes/100 child-weeks seen in children of 30–36 months). The incidence of pneumonia was one-tenth that of ARI and that of severe pneumonia or otitis media was roughly one-tenth that of pneumonia. No case of otitis media was seen in infants less than 2 months of age and none of the toddlers were found to suffer from severe pneumonia.

Table 3 shows the incidence of ARI according to gender. No difference in incidence of ARIs was seen among the two genders. Incidence of pneumonia was, however, noted to be higher in boys (0.9 episodes/100 child-weeks) as compared to girls (0.6 episodes/100 child-weeks). Similar pattern was seen for severe pneumonias also (0.1 for boys as compared to 0.05 for girls). In contrast, the incidence of “no pneumonia, cold, and cough” as well as otitis media was found to be higher in girls. The observed differences were, however, not found to be statistically significant.

**Figure 1:**
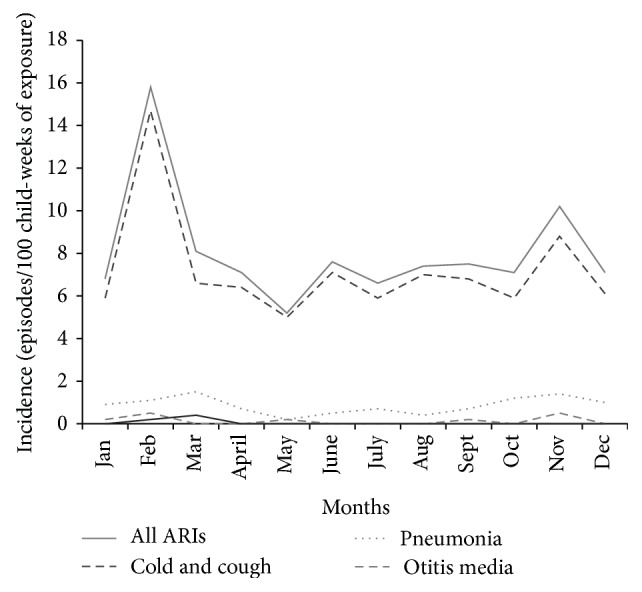
Line diagram showing monthly incidence and trend of ARIs.

Figure 1 shows the monthly incidence of “ARI” (all types including otitis media), “no pneumonia, cough, and cold,” “pneumonia,” and “otitis media” among infants and toddlers. The monthly incidence of ARIs ranged from a low of 5.2 episodes/100 child-weeks (in May) to a high of 15.8 (in February). Two peaks were seen with the more prominent peak falling in the month of February which coincided with spring season. The lesser peak was seen in November, coinciding with autumn season. Incidence of pneumonia also showed a fluctuation, ranging from a low of 0.2 episodes/100 child-weeks in May to a high of 1.5 episodes/100 child-weeks in March (visible as a peak) and November (visible as a less prominent plateau).

## 4. Discussion

This community-based prospective study conducted in Delhi provides information on risk of developing ARI in tropical peri-urban settings of developing countries. Our study had a very low attrition rate (1.6%) all of which was attributable to social migrations. We believe that the child-weeks lost to attrition were not systematically different from those included for analysis. Besides, though living standards of Delhi are better than those in other areas of India as a whole, owing to the lack of primary health care system and higher levels of pollution (as is the case in most urban areas), there is likely to be a balancing effect, thereby making our estimates representative of similar peri-urban areas.

*Prevalence of ARI*. Prevalence of ARI in this study stood at 34.3% as compared to 19.9% and 5.4% for diarrhea and fever, respectively. The relative importance of ARI as a cause of disease, not just mortality, in young children was, hence, reestablished. NFHS 3 [21] reported that 5.8% of children under 5 years suffered from ARI (cough plus short, rapid breathing) during the 2-week-period preceding the survey. Our figures of prevalence of ARI are much higher than that reported by NFHS. The difference is due to variations in methodology (NFHS used a one-time survey technique conducted by nonmedical personnel and no examination was involved). Zaman et al. [22] from neighbouring Bangladesh have also documented ARI to be a major cause of morbidity among rural children with prevalence rates of 35.4 per hundred days of observation. The Bangladesh study, however, reports that nearly 9% of their study subjects did not suffer from any ARI episode during the 12-month study period which is a highly desirable situation compared to only 0.9% in our case. Our findings are also in concurrence with those reported by Koch et al. in their Greenland study [23].

*Incidence of ARI*. Annual incidence of ARI in this study was 4.1 episodes/child (7.9 epi/100 child-wks). Acharya et al. [17] in 2003 found ARI incidence of 6.4 episodes per child per year. Zaman et al. [22] reported 3.7 episodes of ARI among under-five children of Wardha, Maharashtra in 1996, while Awasthi and Pande [13] from Lucknow have reported the lowest ARI incidence of 1.67 episodes. Studies from neighbouring Bangladesh [22] too have reported annual ARI incidence to be 5.5 episodes per child. Although our observations fall within the range of what others have reported, still it would be the best not to compare our findings with that of others for the following three reasons: (i) while most workers have studied children up to 5 years of age, we focused on infants and toddlers exclusively, the age which is most affected by ARIs; (ii) some studies were conducted in part of the year only and not a full calendar year; and (iii) the questions used to estimate ARI might have been different in different studies.

*Incidence of Pneumonia*. We found annual pneumonia incidence to be 0.44 episodes/child (0.85 episodes/100 child-weeks) which is much higher than that reported by most workers and agencies. For example, the joint UNICEF-WHO document on pneumonia of 2006 [6] reports an incidence of 0.30 episodes/child/year for India as a whole and Rudan et al. [24] report the worldwide incidence in developing countries to be 0.28 episodes per year. Singh and Nayar [25] have reported an annual incidence of pneumonia among under-five children of Wardha (Maharashtra) in 1996 to be 0.07 episodes/child, while Awasthi and Pande [13] found pneumonia incidence of 0.09 per child per year in Lucknow, UP. Acharya et al. [17], however, report a higher pneumonia incidence of 0.52 episodes per child per year from rural Udupi in Karnataka. In Bangladesh [22] much lower incidence of acute lower respiratory infections (i.e., pneumonia) was reported (0.23 per child per year).

The reason for the high prevalence of ARIs and higher incidence of pneumonia in the present study could be (i) higher levels of air pollution, (ii) overcrowded and ill-ventilated living conditions, and (iii) lack of a primary health care mechanism. The latter has also been documented in Pakistan by Singh and Nayar [25]which records that 13% of “no pneumonia” is likely to turn into pneumonia, and a significant proportion of these would be prevented if appropriate care is available.

*Pattern of ARIs*. We found 10.1% of all ARI episodes to be pneumonia and 0.7% to be severe pneumonia. Awasthi and Pande [13] found 10% of “respiratory disease” to be “pneumonia” in Lucknow, while Acharya et al. [17] reported 8.7% to be pneumonia and 0.5% to be severe pneumonia in Udupi, Karnataka. In contrast, 4% of ARI episodes were reported to be pneumonia in Bangladesh [22]. On the other hand, incidence of otitis media in the current study (0.01%) is much lower than the 30% estimated incidence among ARI cases as reported by Simoes et al. [12] for developing countries. The difference could be due to difficulty in making an assessment of otitis media which requires otoscopy, a procedure we could not conduct in field conditions.

The mean duration of no pneumonia, cough, and cold was 6.16 days as compared to 11.65 days for pneumonia in our study. The Bangladesh study [22] reported that 46 percent of URI and 65 percent of ALRI episodes lasted 15 days or more without giving any more details. Median duration of URI and LRI episodes has been reported to be 14 days (range: 7–25 days) and 19 days (range: 9–39 days) in the Greenland study [23].

*Age.* Incidence of URI was the highest in the 12–17-month-age cohort, while that for pneumonia was highest in 0-1-month-age cohort. Overall, the incidence of ARI was seen to decline with increasing age. The Bangladesh [22] study found the highest URI incidence among children 18–23 months old, followed by infants 6–11 months old, while the highest pneumonia incidence was reported in infants 0–6 months old, followed by children 12–18 months old. The Greenland study [23] also reported the highest prevalence of respiratory symptoms in the 6–11 months age group with a steep rise occurring from less than 5 months to 6–11 months and a decrease in incidence thereafter. Thus, the risk of ARI generally increases in the later part of life as a toddler, which could be due to the fact that children become more mobile at this age and interact with multiple caregivers and individuals (in contrast to mother only during early infancy) and they are therefore exposed to more numbers of ARI cases/carriers.

*Gender.* Our finding of slightly higher incidence of “no pneumonia, cough, and cold” in girls did not match the findings of NFHS which reported slightly higher prevalence among boys. The reasons for higher reporting for boys could be explained by the gender bias that is prevalent in our society with its attendant preferential treatment for boy-child, especially when history is the only method of assessment (NFHS methodology). The Greenland study [23] did not find any gender differential as far as URIs were concerned, but that for pneumonia was higher in boys. Our findings are in agreement with the Greenland study.

*Seasonality*. The presentstudydemonstrated that URI peaks in spring season and to a lesser extent in autumn, while pneumonia peaks a little later. The Bangladesh [22] study noted a higher incidence of URI as well as pneumonia (ALRI) in monsoon and autumn (before winter). On the other hand, no seasonal pattern in the incidence of ARI, URI, or LRI was observed in the Greenland study [23]. The differences are possibly due to differences in the climatic conditions as well as housing conditions of the areas of study. No Indian study could be found with which we could compare our findings.

## 5. Conclusion and Recommendations

The annual incidence of ARIs was 4.1 episodes/child, while that for pneumonia was 0.44 episodes/child. Pneumonia incidence was higher than the estimates for India as well as for South Asia as a whole. Incidence of pneumonia was roughly one-tenth that of ARI and that of severe pneumonia or otitis media was one-tenth that of pneumonia. Since incidence of ARIs and pneumonia generally decreased with increasing age, targeting infants specifically for prevention and control efforts may be a more effective strategy. Two fortnight-long intensive mass-media campaigns (*Pneumonia Awareness and Prevention Fortnight*) prior to Holi (a festival of colors celebrated in the month of March commencing the spring season) and Dusshera (Indian festival celebrated in autumn during month of October/November ), on the lines of “Breast Feeding Awareness Week” and “Malaria Month,” just before onset of the peak season, are also likely to be helpful in generation awareness and disease control. More of such studies with larger sample sizes and including rural as well as urban populations need to be conducted.

## 6. Limitations

There are some limitations of this study. The most prominent of these are as follows. (i) With the study being conducted in a small area, its findings may not be generalizable to other peri-urban settings, within and outside this country. (ii) Period of recall of one fortnight, though accepted as standard for this type of study, may have led to missing out on many episodes (those starting after a particular visit and ending before the next), particularly when primary caregiver was not the respondent. (iii) Primary researcher being a doctor was ethically bound to provide health education as well as medical guidance to children and their caregivers, thereby altering the natural history and may be even the incidence of ARIs. All these limitations might have underestimated the burden of ARI to some extent.

## Figures and Tables

**Table 1:** Sociodemographic, environmental, and health characteristics of the study subjects.

Characteristic (*N* = 106)	Number (%)
(1) Age at enrolment	
0-1 month	29 (27.4)
2–11 months	38 (35.8)
12–35 months	39 (36.8)
(2) Type of family	
Nuclear	31 (29.2)
Joint	75 (70.8)
(3) Socioeconomic status of the family∗	
Middle	84 (79.2)
Upper lower	22 (20.8)
(4) Mother's literacy status	
Up to primary	13 (12.3)
Middle/high school	54 (51.0)
Higher secondary	16 (15.0)
Graduate/higher	23 (21.7)
(5) Overcrowding	
Present	50 (47.2)
(6) Natural ventilation	
Inadequate	22 (20.8)
(7) Separate kitchen	
Present	91 (85.8)
(8) Fuel used for cooking	
LPG only	87 (82.1)
Biomass + LPG	19 (17.9)
(9) Exposure to ETS	
Present	48 (45.3)
(10) Prevalence of malnutrition	
Mild to moderate	27 (23.6)

^*^Assessed by modified Kuppuswamy scale with price index corrected for 2011.

**Table 2:** Incidence, clinical pattern, and severity of ARI in infants and toddlers.

Age cohort (child-weeks of exposure in age cohort, *N*)	Number of episodes (incidence per 100 child-weeks)				
	No pneumonia, cold, and cough^#^	Pneumonia∗	Severe pneumonia	Otitis media	Any ARI
0-1 (118)	10 (8.5)	2 (1.7)	2 (1.7)	—	12 (10.2)
2–5 (652)	49 (7.5)	6 (0.9)	1 (0.2)	1 (0.2)	55 (8.4)
6–11 (1505)	129 (8.6)	17 (1.1)	—	1 (0.001)	146 (9.7)
12–17 (1207)	83 (6.9)	9 (0.8)	—	1 (0.001)	92 (7.6)
18–23 (1098)	72 (6.6)	7 (0.6)	—	1 (0.001)	79 (7.2)
24–29 (680)	39 (5.7)	4 (0.6)	—	1 (0.001)	43 (6.3)
30–35 (243)	8 (3.3)	2 (0.8)	—	—	10 (4.1)
All ages (5503)	390 (7.1)	47 (0.85)	3 (0.054)	(0.09)	437 (7.9)

^*^Including severe pneumonia, ^#^including otitis media; *P* = 0.0004, *χ* ^2^ = 45.404, and df = 18.

Statistically significant for under 2 months children having ARI as compared to children with ARI from other age groups.

**Table 3:** Incidence of ARI according to gender.

Gender (child-weeks of exposure in cohort, *N*)	Number of episodes (incidence per 100 child-weeks)				
	All ARIs	No pneumonia, cold, and cough	Pneumonia	Severe pneumonia	Otitis media
Boys (3475)	277 (7.9)	243 (6.9)	34 (0.9)	2 (0.1)	3 (0.09)
Girls (2028)	160 (7.9)	147 (7.1)	13 (0.6)	1 (0.05)	2 (0.10)
All children (5503)	437 (7.9)	390 (7.0)	47 (0.8)	3 (0.1)	5 (0.09)

*P* = 0.6025, *χ* ^2^ = 1.857, df = 3.

Chi-square test is statistically nonsignificant when compared for “pneumonia” between boys and girls.
